# Multi‐cohort analysis identifies somatic *NTRK* mutations as a biomarker for immune checkpoint inhibitor use in cutaneous melanoma

**DOI:** 10.1002/ctm2.1478

**Published:** 2023-11-21

**Authors:** Junya Yan, Long Deng, Jiayi Yu, Xiaowen Wu, Shaoyu Wang, Shundong Cang

**Affiliations:** ^1^ Department of Oncology Henan Provincial People's Hospital, Zhengzhou University People's Hospital, Henan University People's Hospital Zhengzhou China; ^2^ Western Medical Branch of the Chinese PLA General Hospital Beijing China; ^3^ Department of Radiation Oncology Key Laboratory of Carcinogenesis and Translational Research (Ministry of Education/Beijing) Peking University Cancer Hospital & Institute Beijing China; ^4^ Department of Renal Cancer and Melanoma Key Laboratory of Carcinogenesis and Translational Research (Ministry of Education/Beijing) Peking University Cancer Hospital & Institute Beijing China; ^5^ Henan Key Laboratory of Imaging and Intelligent Processing PLA Strategic Support Force Information Engineering University Zhengzhou China

Dear Editor,

Although immune checkpoint inhibitor (ICI) therapy has revolutionised the treatment approach for various types of cancer, including cutaneous melanoma,^1^ the proportion of patients who experience durable responses to ICI therapy remains limited.^2^ This study aimed to identify novel biomarkers that could discern individuals who respond favorably to ICI therapy, as well as to facilitate the development of alternative treatment options for non‐responders.

*NTRK* genes, specifically *NTRK1*, *NTRK2* and *NTRK3*, are situated in distinct regions of chromosome 1q22, 9q21 and 15q25, respectively.^3^ They encode transmembrane proteins known as tyrosine protein kinases, namely, TRKA, TRKB and TRKC. In normal cellular contexts, TRK proteins are capable of regulating essential cellular processes through the activation of various downstream pathways, including SHC, FRS2, PLCγ, MAPK, PI3K and PKC.^4^ Recently, numerous studies have documented the involvement of single nucleotide variants, gene fusions, gene overexpression and other aberrations within the TRK pathway in the pathogenesis of cancers. Interestingly, colorectal cancer harbouring *NTRK* fusion defines a unique subset with high tumour mutation burden (TMB) and microsatellite instability.^5^ Evidence has also demonstrated a positive association between NTRK3 expression and diverse immune lymphocytes in patients with bladder cancer.^6^ These findings suggested a correlation between *NTRK* alterations and the tumour immune microenvironment to a certain extent. Considering that somatic *NTRK* mutations have been identified in several types of cancer,^7^, ^8^, ^9^, ^10^ we aimed to elucidate the prognostic role of somatic *NTRK* mutations and their association with the efficacy of ICI therapy in cutaneous melanoma.

The flowchart of this study is presented in Figure S1. We first investigated the incidence of somatic *NTRK* mutations in pan‐tumour samples from The Cancer Genome Atlas (TCGA) database. Notably, patients with cutaneous melanoma exhibited the highest prevalence of somatic *NTRK* mutations (19.5%, 86/440) compared to other 32 types of cancer (Figure 1A). The somatic mutation rates for *NTRK1*, *NTRK2* and *NTRK3* in cutaneous melanoma are 8%, 4% and 11%, respectively (Figure 1B). Among all 111 mutation cases, the frequencies of missense mutation, truncating mutation and splice mutation are 89%, 8% and 3%, respectively (Figure S2 and Table S1). Additionally, only two cases of missense mutation are with driver significance (Table S1). Survival analysis showed that the median overall survival (mOS) time for cutaneous melanoma patients harbouring somatic *NTRK* mutations (*NTRK* ^Mut^) was relatively shorter than that for patients with wild‐type *NTRK* (*NTRK* ^WT^) (54.4 vs. 95.0 months; *p* = .061; Figure 1C), although the difference did not reach a significance. We further investigated the relationship between somatic *NTRK* mutation and TMB. Interestingly, cutaneous melanoma patients with *NTRK* ^Mut^ harboured markedly higher TMB compared to *NTRK* ^WT^ patients (Figure 1D), indicating the potential influence of somatic *NTRK* mutations on the efficacy of ICI therapy in cutaneous melanoma.

**FIGURE 1:**
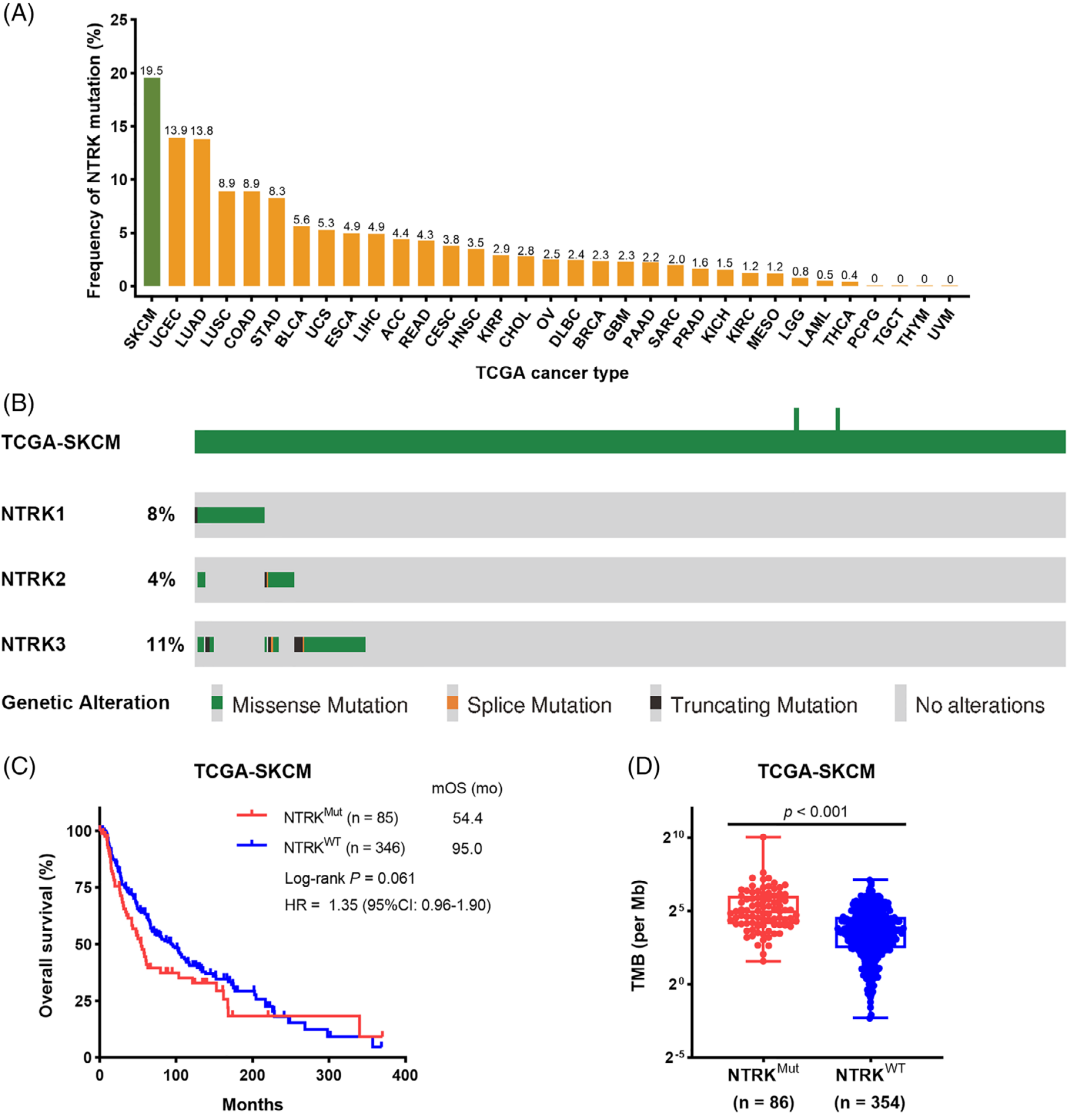
Prevalence of somatic *NTRK* mutations in cutaneous melanoma. (A) The proportion of somatic *NTRK* mutated tumours identified for each cancer type in The Cancer Genome Atlas (TCGA) pan‐cancer cohorts. (B) Mutation patterns (as Oncoprint schematics) across the *NTRK* family hyper‐altered patients from the TCGA‐skin cutaneous melanoma (SKCM) cohort. (C) Prognostic value of somatic *NTRK* mutations in cutaneous melanoma. (D) Comparison of the tumour mutation burden (TMB) levels between somatic *NTRK* mutations (*NTRK* ^Mut^) and wild‐type *NTRK* (*NTRK* ^WT^) subgroups. CI, confidence interval; HR, hazard ratio; mOS, median overall survival.

We next aimed to explore the relationship between somatic *NTRK* mutations and the clinical benefit of ICI therapy in melanoma. The discovery cohort included 435 melanoma patients (77% are cutaneous subtype) from four reported ICI‐treated datasets (Table S2). In the discovery cohort, *NTRK* ^Mut^ prevailed in 12.9% patients with melanoma and conferred significantly better objective response rate (ORR) (42.8% vs. 23.5%; *p* = .002; Figure 2A), disease control rate (DCR) (57.1% vs. 40.1%; *p* = .012; Figure 2A) and longer overall survival (OS) (34.5 vs. 21.7 months; *p* = .017; Figure 2B). The pooled analysis indicated that no significant heterogeneity among the four datasets was observed regarding the trend of prolonged OS in *NTRK* ^Mut^ patients (*I* ^2^ = 0, *p* = 1.00; Figure 2C). We also conducted multivariate Cox analysis and demonstrated that somatic *NTRK* mutation status, along with sex, M stage, lactate dehydrogenase level and ICI therapy regimen, independently influenced OS (Figure 2D).

**FIGURE 2:**
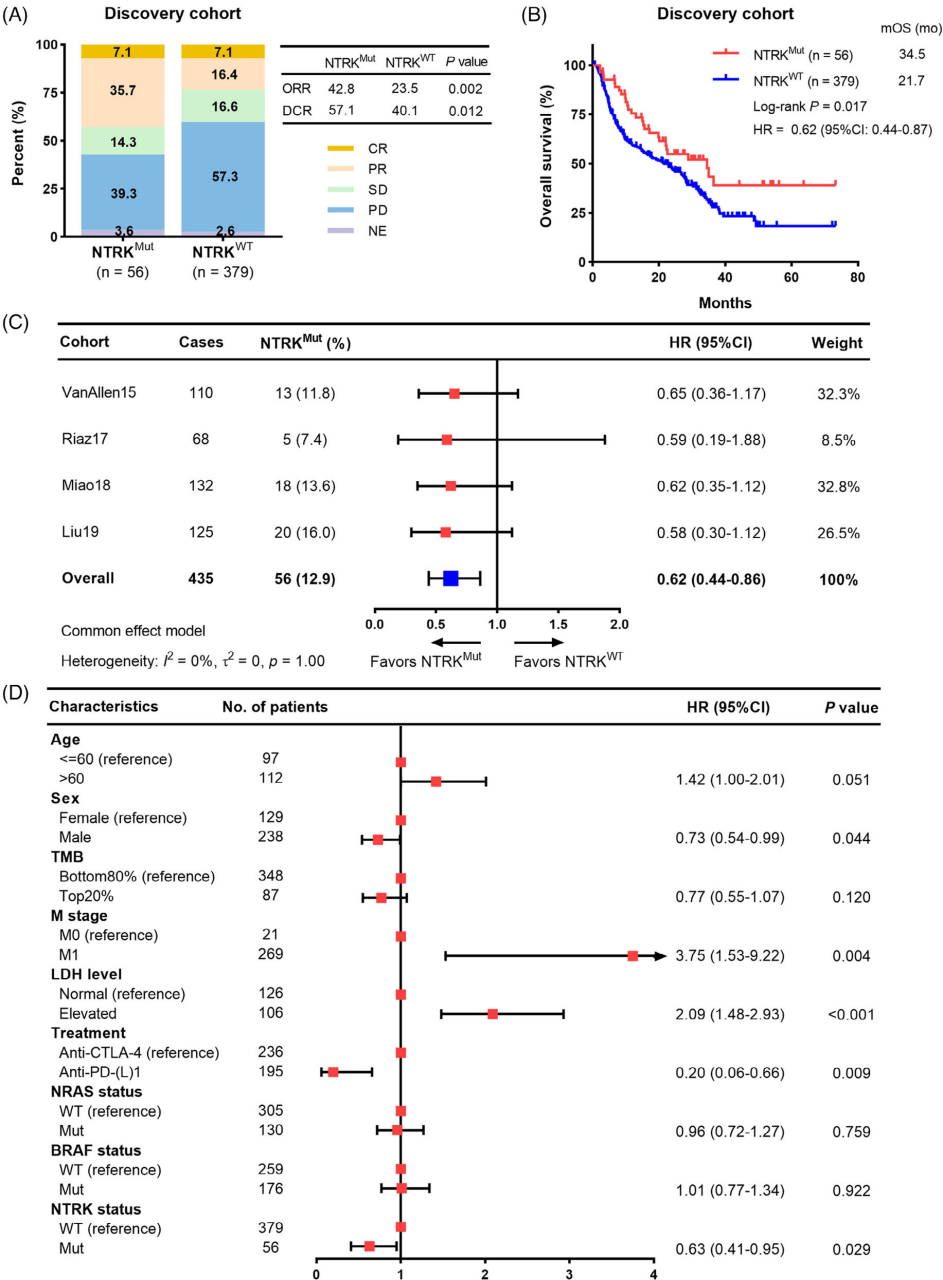
Exploration of the association between somatic *NTRK* mutations and clinical benefit for immune checkpoint inhibitor (ICI) therapy in the discovery cohort. (A) The ratio of patients with complete response (CR), partial response (PR), stable disease (SD), progressive disease (PD), and not evaluated (NE) treated with ICI therapy in *NTRK* mutations (*NTRK* ^Mut^) and wild‐type *NTRK* (*NTRK* ^WT^) subgroups. (B) Kaplan–Meier survival curves of overall survival comparing the *NTRK* ^Mut^ and *NTRK* ^WT^ subgroups. (C) Forest plot of *NTRK* ^Mut^ with longer overall survival in patients from four datasets. (D) Multivariate Cox regression analyses of NTRK status and clinicopathologic factors associated with overall survival. DCR, disease control rate; ORR, objective response rate.

To validate the association between somatic *NTRK* mutations and clinical benefit from ICI therapy in melanoma, we further included the Memorial Sloan Kettering Cancer Center (MSKCC) cohort, which consisted of 1661 patients with >10 types of cancer undergoing ICI therapy for subsequent analysis (Table S2). In MSKCC‐melanoma subgroup, *NTRK* ^Mut^ patients exhibited significantly prolonged OS compared to *NTRK* ^WT^ patients (mOS: not reached vs. 42.0 months; *p* = .040; Figure 3A). However, the association between somatic *NTRK* mutations and OS in non‐small cell lung cancer (Figure 3B), colorectal cancer (Figure 3C), bladder cancer (Figure 3D), head and neck cancer (Figure 3E) and cancer of unknown primary (Figure 3F) was not significant. These data suggested that somatic *NTRK* mutations may serve as a predictor of improved response rates and survival outcomes in the context of ICI therapy only for melanoma but not for pan‐cancer, which warranted further exploration and larger samples to validate.

**FIGURE 3:**
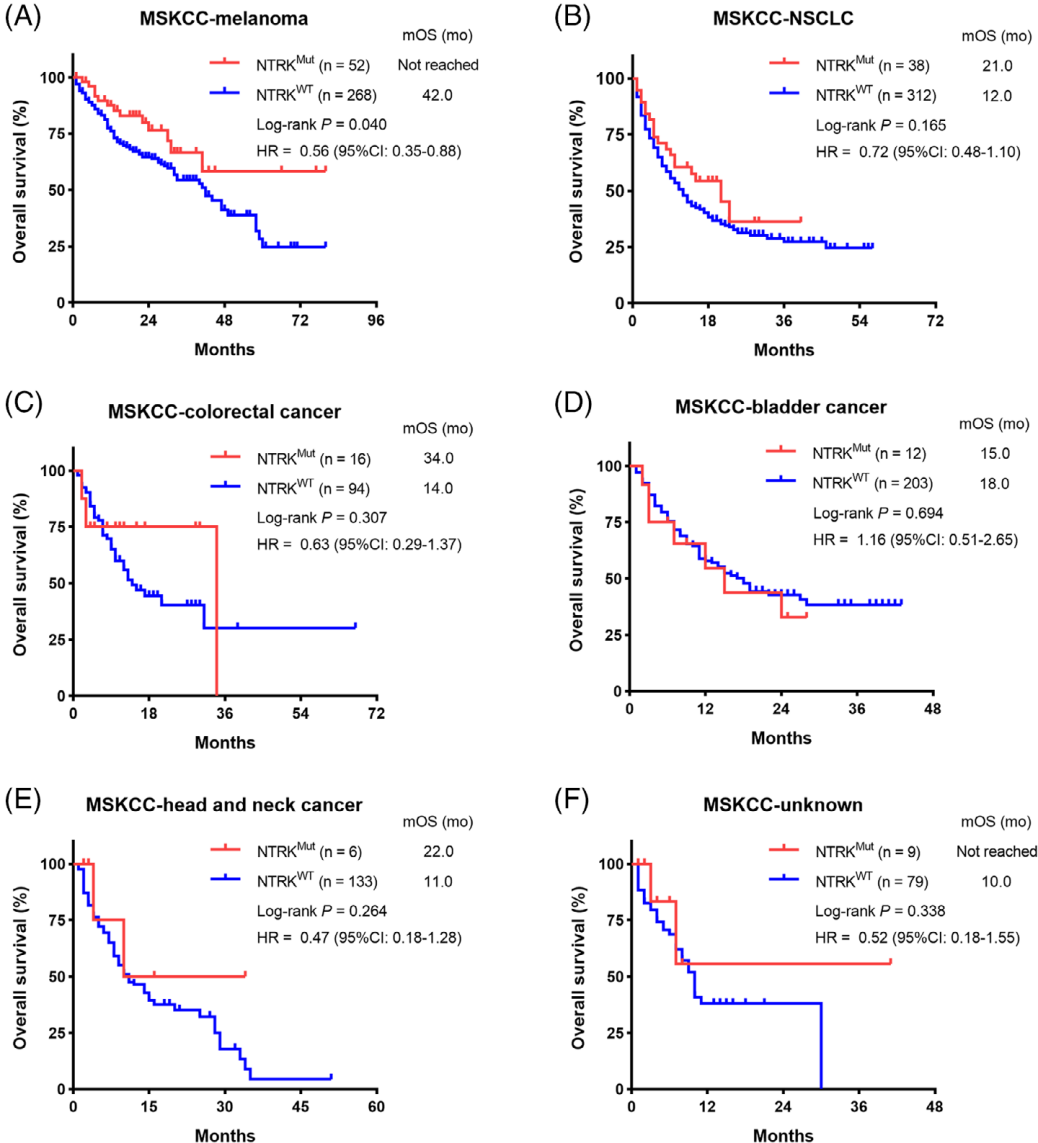
Validation of the predictive value of somatic *NTRK* mutations for immune checkpoint inhibitor (ICI) therapy in MSKCC pan‐cancer cohorts. Kaplan–Meier curves of overall survival between the *NTRK* mutations (*NTRK* ^Mut^) and wild‐type *NTRK* (*NTRK* ^WT^) subgroups in melanoma (A), non‐small cell lung cancer (NSCLC) (B), colorectal cancer (C), bladder cancer (D), head and neck cancer (E), and cancer of unknown primary (F).

The discovery cohort and MSKCC‐melanoma cohort were integrated to form one pooled cohort (*n* = 755). Correlation analysis showed that somatic *NTRK* mutations were associated with higher TMB in the pooled cohort (Figures S3A and S4), which was consistent with the TCGA‐skin cutaneous melanoma cohort. Additionally, patients with TMB‐high and *NTRK* ^Mut^ harboured a significantly longer OS compared to those with TMB‐low and *NTRK* ^WT^ (Figure S3B,C). These results revealed that the combined utilization of somatic *NTRK* mutations and TMB could accurately identify advantageous populations for ICI therapy in melanoma.

Considering that the ICI therapy regimen was also a prognostic factor for melanoma patients, we also explored the influence of somatic *NTRK* mutations on drug selection for ICI therapy. For *NTRK* ^Mut^ patients in the pooled cohort, no significant survival outcome difference was observed between the anti‐programmed death‐(ligand) 1 [anti‐PD‐(L)1] and anti‐cytotoxic T‐lymphocyte antigen 4 (anti‐CTLA‐4) groups (Figure S5A). In contrast, anti‐PD‐(L)1 group exhibited markedly longer OS compared to anti‐CTLA‐4 group in *NTRK* ^WT^ patients (Figure S5B). Collectively, somatic *NTRK* mutations may exert a protective role for ICI therapy regardless of the specific regimen, while the efficacy of anti‐PD‐(L)1 might be superior to that of anti‐CTLA‐4 for *NTRK* ^WT^ melanomas.

In summary, our study first demonstrated that somatic *NTRK* mutations might act as a novel predictor for the efficacy of ICI therapy in cutaneous melanoma, and that its combination utilization with TMB may improve the stratification accuracy of patients for ICI therapy. In addition, the mutation status of *NTRK* may influence the drug options of ICI therapy in cutaneous melanoma, which warrants further investigations in prospective clinical trials (Figure 4).

**FIGURE 4:**
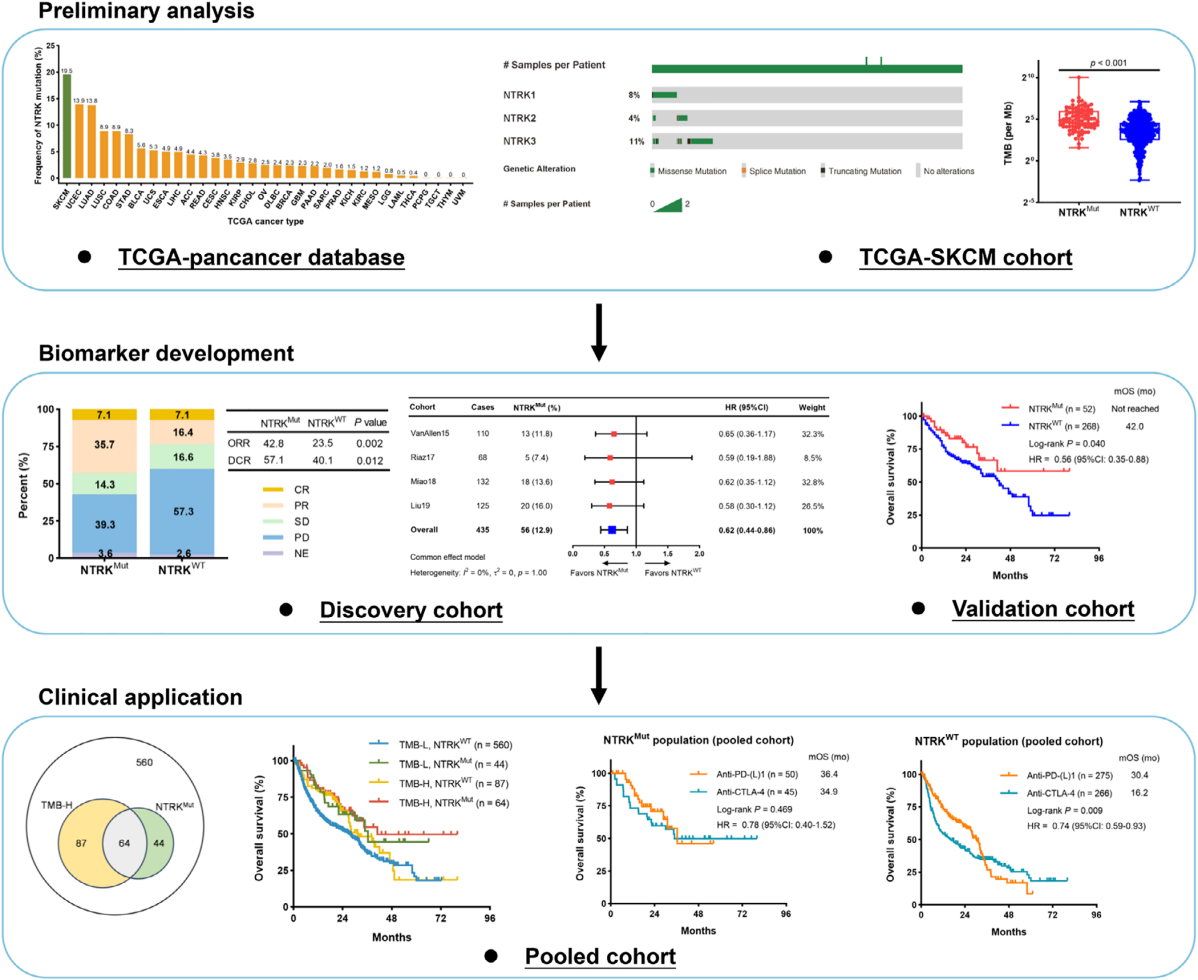
Graphical abstract for immune characterization of somatic *NTRK* mutations in cutaneous melanoma.

## AUTHOR CONTRIBUTIONS

Shundong Cang and Junya Yan designed this study. Junya Yan, Long Deng, and Jiayi Yu collected the clinical and sequencing data. Junya Yan, Long Deng, Jiayi Yu, Xiaowen Wu, and Shaoyu Wang performed the analysis of data. Junya Yan and Long Deng write the first draft of the manuscript. All authors read and approved the final manuscript.

## CONFLICT OF INTEREST STATEMENT

The authors declare they have no conflicts of interest.

## ETHICAL APPROVAL

Not applicable.

## Supporting information

Supporting Information

## Data Availability

All the data and materials are available within the paper.
