# Supplementary material for: Multi‐cohort analysis identifies somatic NTRK mutations as a biomarker for immune checkpoint inhibitor use in cutaneous melanoma
Source: Clin Transl Med. 2023 Nov 21;13(11):e1478. doi: 10.1002/ctm2.1478 (PMC10660794; doi:10.1002/ctm2.1478)
Supplement: Supplementary file 1 — Supporting Information [file CTM2-13-e1478-s001.docx]

**Supplementary Materials**

**Multi-cohort analysis identifies somatic *NTRK* mutations as a biomarker for immune checkpoint inhibitor use in cutaneous melanoma**

Junya Yan, Long Deng, Jiayi Yu, Xiaowen Wu, Shaoyu Wang, Shundong Cang

Supplementary Methods……………………………………………………………….2

Supplementary Figure S1………………………………………………………………5

Supplementary Figure S2………………………………………………………………6

Supplementary Figure S3………………………………………………………………7

Supplementary Figure S4………………………………………………………………8

Supplementary Figure S5………………………………………………………………9

Supplementary Table S1……………………………………………………………...10

Supplementary Table S2……………………………………………………………...13

**Supplementary Methods**

**Somatic *NTRK* mutations**

The non-synonymous mutations including TRUNC (Frameshift del, Frameshift ins, nonsense, nonstop, splice region, splice site), INFRAME (Inframe del and Inframe ins) and MISSENSE mutations of at least one *NTRK* subtype (including *NTRK1*, *NTRK2*, and *NTRK3*) were defined as *NTRK*^Mut^ in this study.

**The Cancer Genome Atlas (TCGA) cohort**

Somatic mutations of 10387 patients across 33 tumor types, including skin cutaneous melanoma (SKCM), were retrieved from the cBioPortal for Cancer Genomics (https://www.cbioportal.org/), which were used for analysis of *NTRK*^Mut^ incidence and mutation distribution. The MutationMapper module (https://www.cbioportal.org/mutation_mapper) from the cBioPortal was used to investigate the distribution of mutations in the protein domain. The survival data of 431 patients with melanoma were extracted from TCGA-SKCM cohort was used to explore the prognostic influence of *NTRK* mutation status.

**Clinical discovery and validation cohorts**

To evaluate the predictive value of somatic *NTRK* mutations in immune checkpoint inhibitors (ICI)-treated melanoma, we systematically collected the genome sequencing data and corresponding clinical information from four publicly available cohorts treated with anti-PD-(L)1 or (and) anti-CTLA-4 therapy (Table S2), including VanAllen15 (n = 110), Riaz17 (n = 68), Miao18 (n = 132), and Liu19 (n = 125). These four cohorts together constituted the discovery cohort which comprised 435 patients with melanoma. Furthermore, we validated the predictive potency of somatic *NTRK* mutations in the MSKCC cohort comprised 1661 patients with >10 types of tumors treated with anti-CTLA-4 or (and) anti-PD-(L)1 therapy (Table S2).

For the VanAllen15, Miao18, Liu19, and MSKCC cohorts, the information regarding clinical and sequencing data were downloaded from cBioPortal for Cancer Genomics (https://www.cbioportal.org/). For the Riaz17 cohort, the clinical and sequencing data were obtained from the published article.

**Clinical outcomes**

Objective response rate (ORR), disease control rate (DCR), and overall survival (OS) were the primary clinical outcomes. Patient response was determined using the Response Evaluation Criteria in Solid Tumors (RECIST) version 1.1 criteria (complete response, CR; partial response, PR; stable disease, SD; progressive disease, PD), and the ORR was defined as the proportion of patients with CR or PR, and the DCR was defined as the proportion of patients with CR or PR or SD. The OS was calculated from the start data of ICI therapy to the date of death. Patients who did not die were censored on the date of their last scan.

**Tumor mutation burden (TMB) analysis**

TMB was defined as the total number of mutations per megabase (Mb) of genome with non-synonymous somatic, coding, base substitution, and indel variants and was calculated using maftools. We separated the TMB data of TCGA-SKCM and five ICI-treated cohorts and investigated its correlation to *NTRK* mutation status.

**Statistical analyses**

The SPSS software (version 23.0) and GraphPad Prism software (version 8) were used for statistical analyses and graphical drawing. Continuous variables were compared by Mann-Whitney U test and categorical variables were compared by chi-square test or Fisher’s exact test. The Kaplan–Meier curve analysis of OS was compared by the log-rank test. The Cox proportional hazards regression was applied for multivariate analysis, and available confounding factors including age, sex, TMB, M stage, lactate dehydrogenase, ICI treatment, *NRAS* mutation status, and *BRAF* mutation status were adjusted. All reported *P*-values were two-tailed, and *P* < 0.05 was considered statistically significant. Q-test and I^2^ statistics were used to assess the heterogeneity. A result of *P* > 0.1 and I^2^ < 50% indicated no significance between-study heterogeneity.


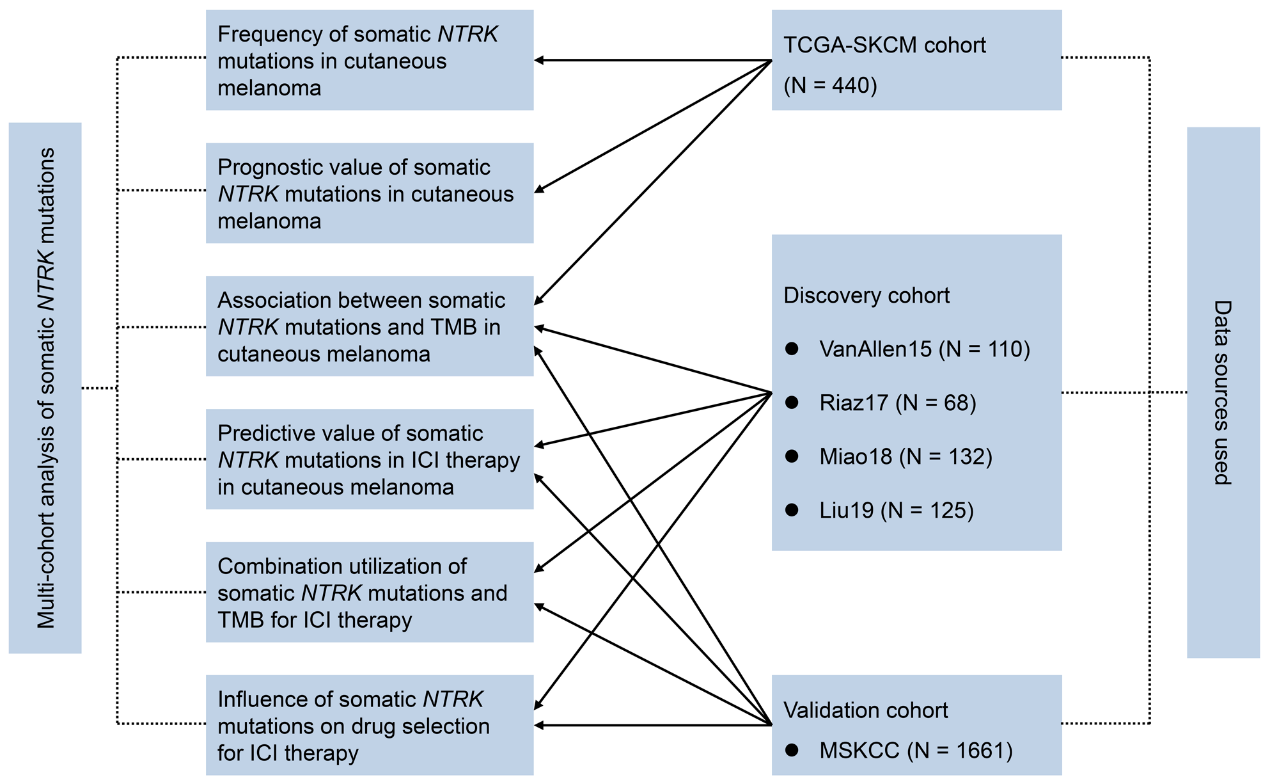


**Supplementary Figure S1.** The flow diagram of this study. The connected arrow between analysis aim (middle left) and data source (middle right) means the used cohort by this analysis.


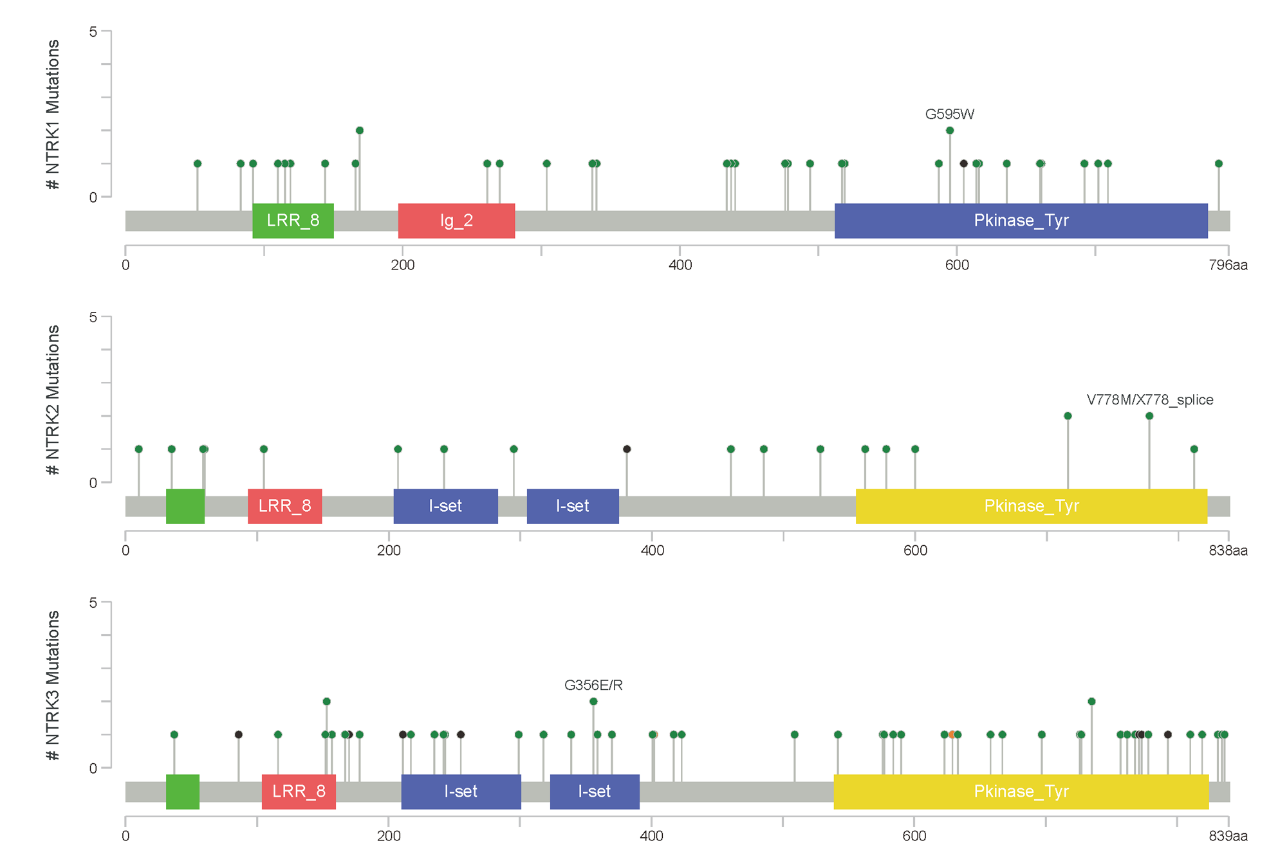


**Supplementary Figure S2.** Lollipop plot showing the loci distribution of mutations across the *NTRK* family hyper-altered patient cohorts from the TCGA-SKCM cohort.


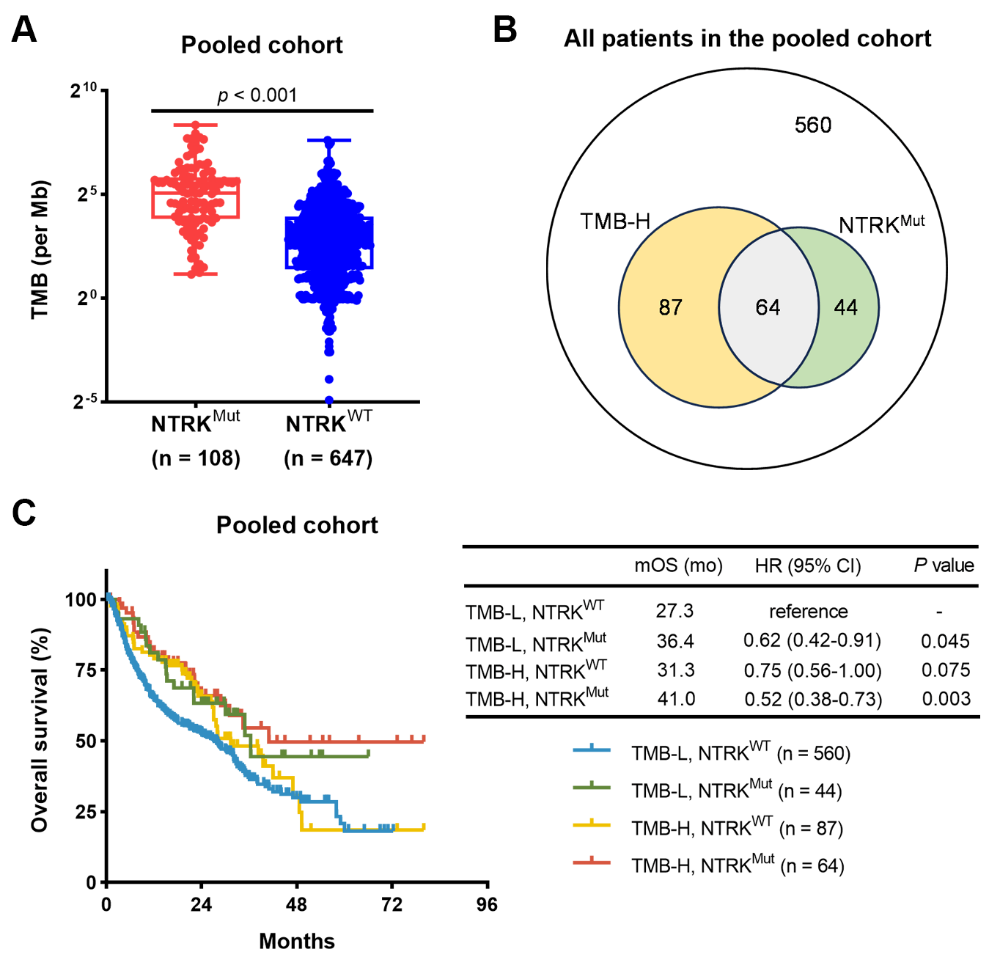


**Supplementary Figure S3.** The association between somatic *NTRK* mutations and TMB. A, Comparison of the TMB level between *NTRK*^Mut^ and *NTRK*^WT^ subgroups in the pooled cohort. B, The Venn diagram showing the concomitant presence of TMB-H and *NTRK*^Mut^ in the pooled cohort. C, Kaplan-Meier curves of overall survival among four subgroups according to TMB and *NTRK* status.


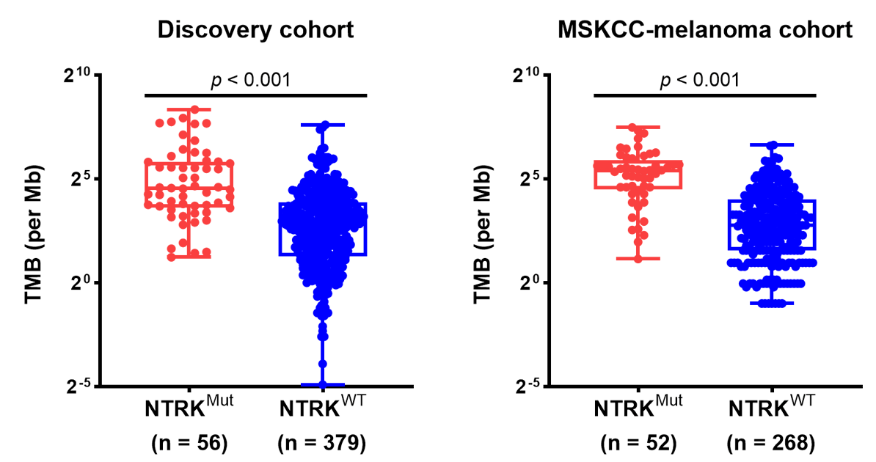


**Supplementary Figure S4.** Comparison of the TMB level between *NTRK*^Mut^ and *NTRK*^WT^ subgroups in the discovery cohort and MSKCC-melanoma cohort.


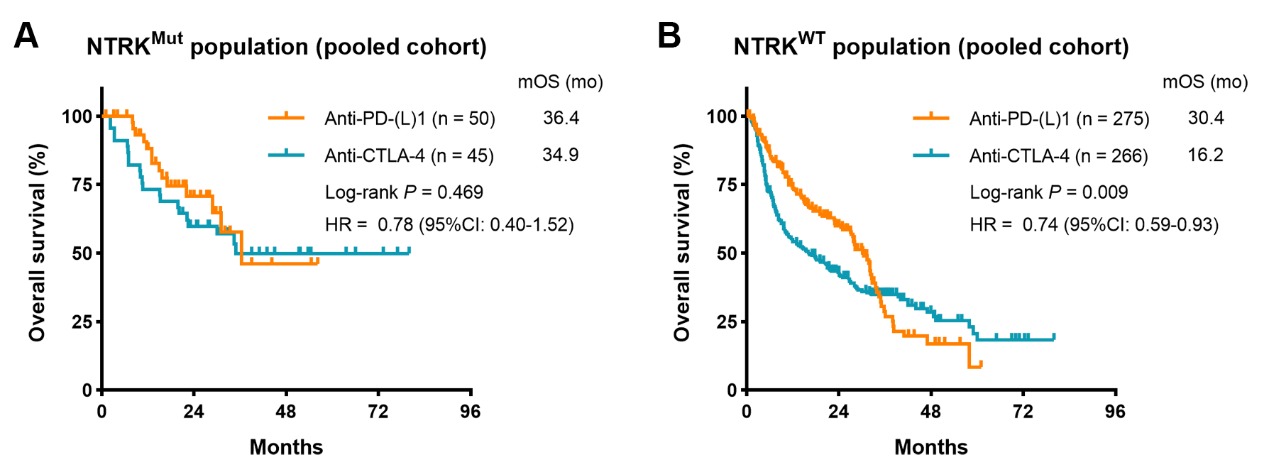


**Supplementary Figure S5.** Difference in overall survival between melanoma patients receiving anti-CTLA-4 or anti-PD-(L)1 therapy. A-B, Kaplan-Meier curves of overall survival between anti-PD-(L)1 and anti-CTLA-4 subgroups in *NTRK*^Mut^ (A) and *NTRK*^WT^ (B) population from the pooled cohort, respectively.

**Supplementary Table S1.** Detailed information of somatic *NTRK* mutations of 440 samples in TCGA-SKCM cohort.

| **Gene** | **Sample ID** | **Protein Change** | **Mutation Type** | **Variant Type** | **Significance Type** |
| --- | --- | --- | --- | --- | --- |
| NTRK1 | TCGA-EE-A17Y-06  TCGA-ER-A19F-06  TCGA-ER-A19M-06  TCGA-D3-A2JK-06  TCGA-Z2-A8RT-06  TCGA-EE-A2GI-06  TCGA-EB-A3Y6-01  TCGA-EE-A3AD-06  TCGA-EB-A5FP-01  TCGA-BF-A3DL-01  TCGA-EE-A3J5-06  TCGA-EE-A3JA-06  TCGA-ER-A19E-06  TCGA-EB-A3Y7-01  TCGA-BF-A1PU-01  TCGA-EE-A2MR-06  TCGA-FS-A4FC-06  TCGA-EE-A17X-06  TCGA-DA-A1I7-06  TCGA-GF-A6C9-06  TCGA-FW-A3R5-06  TCGA-FW-A3R5-06  TCGA-ER-A193-06  TCGA-D3-A2JC-06  TCGA-D3-A2JD-06  TCGA-D3-A8GI-06  TCGA-EE-A2GE-06  TCGA-FR-A729-06  TCGA-GN-A8LK-06  TCGA-D3-A3C6-06  TCGA-D3-A8GV-06  TCGA-EE-A29X-06  TCGA-GN-A262-06  TCGA-HR-A2OG-06  TCGA-W3-A828-06  TCGA-W3-AA1V-06 | G595W  G595W  R692C  G169R  G169R  K261E  D616N  T340I  S304F  T434M  A110V  R702C  Q660L  R119P  A636V  D709N  N440S  G661E  A337T  G166R  E92K  G519S  G517R  L476M  R52L  M587I  P115H  D270N  E83K  P789T  V144I  P478T  G605  G614V  L437F  P494S | Missense_Mutation  Missense_Mutation  Missense_Mutation  Missense_Mutation  Missense_Mutation  Missense_Mutation  Missense_Mutation  Missense_Mutation  Missense_Mutation  Missense_Mutation  Missense_Mutation  Missense_Mutation  Missense_Mutation  Missense_Mutation  Missense_Mutation  Missense_Mutation  Missense_Mutation  Missense_Mutation  Missense_Mutation  Missense_Mutation  Missense_Mutation  Missense_Mutation  Missense_Mutation  Missense_Mutation  Missense_Mutation  Missense_Mutation  Missense_Mutation  Missense_Mutation  Missense_Mutation  Missense_Mutation  Missense_Mutation  Missense_Mutation  Nonsense_Mutation  Missense_Mutation  Missense_Mutation  Missense_Mutation | SNP  SNP  SNP  SNP  SNP  SNP  SNP  SNP  SNP  SNP  SNP  SNP  SNP  SNP  SNP  SNP  SNP  SNP  SNP  SNP  SNP  SNP  SNP  SNP  SNP  SNP  SNP  SNP  SNP  SNP  SNP  SNP  SNP  SNP  SNP  SNP | putative driver  putative driver  unknown significance  unknown significance  unknown significance  unknown significance  unknown significance  unknown significance  unknown significance  unknown significance  unknown significance  unknown significance  unknown significance  unknown significance  unknown significance  unknown significance  unknown significance  unknown significance  unknown significance  unknown significance  unknown significance  unknown significance  unknown significance  unknown significance  unknown significance  unknown significance  unknown significance  unknown significance  unknown significance  unknown significance  unknown significance  unknown significance  unknown significance  unknown significance  unknown significance  unknown significance |
| NTRK2 | TCGA-FW-A3TV-06  TCGA-GN-A266-06  TCGA-EE-A3JI-06  TCGA-EE-A181-06  TCGA-EE-A29T-06  TCGA-EB-A44N-01  TCGA-EE-A29D-06  TCGA-EE-A17X-06  TCGA-D9-A4Z6-06  TCGA-EB-A6R0-01  TCGA-FW-A3R5-06  TCGA-FW-A3R5-06  TCGA-EE-A3AA-06  TCGA-D3-A8GI-06  TCGA-ER-A19P-06  TCGA-BF-A1Q0-01  TCGA-D3-A1Q1-06  TCGA-D3-A3CE-06  TCGA-EE-A2MQ-06  TCGA-W3-AA1V-06 | E562K  E242K  P295S  D716N  D600N  E528K  S35F  M812I  T207I  E59Q  P10T  V778M  W381  S485F  D716E  X778_splice  P578T  S460Y  P60H  F105L | Missense_Mutation  Missense_Mutation  Missense_Mutation  Missense_Mutation  Missense_Mutation  Missense_Mutation  Missense_Mutation  Missense_Mutation  Missense_Mutation  Missense_Mutation  Missense_Mutation  Missense_Mutation  Nonsense_Mutation  Missense_Mutation  Missense_Mutation  Splice_Site  Missense_Mutation  Missense_Mutation  Missense_Mutation  Missense_Mutation | SNP  SNP  SNP  SNP  SNP  SNP  SNP  SNP  SNP  SNP  SNP  SNP  SNP  SNP  SNP  SNP  SNP  SNP  SNP  SNP | unknown significance  unknown significance  unknown significance  unknown significance  unknown significance  unknown significance  unknown significance  unknown significance  unknown significance  unknown significance  unknown significance  unknown significance  unknown significance  unknown significance  unknown significance  unknown significance  unknown significance  unknown significance  unknown significance  unknown significance |
| NTRK3 | TCGA-EE-A2GU-06  TCGA-GN-A266-06  TCGA-EB-A5SH-06  TCGA-EE-A3JI-06  TCGA-GF-A3OT-06  TCGA-EE-A29E-06  TCGA-EE-A3AG-06  TCGA-EB-A44N-01  TCGA-EE-A2GD-06  TCGA-FR-A726-01  TCGA-EE-A29D-06  TCGA-D3-A5GO-06  TCGA-FR-A69P-06  TCGA-EE-A29H-06  TCGA-EE-A29M-06  TCGA-EE-A2MR-06  TCGA-EE-A29L-06  TCGA-FR-A3YO-06  TCGA-EE-A20H-06  TCGA-EE-A2MD-06  TCGA-FW-A3R5-06  TCGA-EE-A29V-06  TCGA-FW-A3R5-06  TCGA-FW-A3R5-06  TCGA-EE-A182-06  TCGA-DA-A3F8-06  TCGA-D3-A2JF-06  TCGA-D3-A2JF-06  TCGA-FW-A3TU-06  TCGA-EE-A3AG-06  TCGA-3N-A9WC-06  TCGA-BF-A1PX-01  TCGA-D3-A8GB-06  TCGA-D3-A8GC-06  TCGA-D3-A8GI-06  TCGA-D3-A8GN-06  TCGA-EE-A2GH-06  TCGA-EE-A2M8-06  TCGA-RP-A695-06  TCGA-W3-A825-06  TCGA-YD-A89C-06  TCGA-Z2-A8RT-06  TCGA-D3-A2JD-06  TCGA-D3-A2JK-06  TCGA-DA-A95Y-06  TCGA-EE-A183-06  TCGA-EE-A2ME-06  TCGA-EE-A3AA-06  TCGA-ER-A19P-06  TCGA-FR-A8YC-06  TCGA-GF-A3OT-06  TCGA-GN-A263-01  TCGA-GN-A26D-06  TCGA-W3-AA1V-06  TCGA-YD-A89C-06 | G623E  Q773  G235E  V726L  M667I  G356R  V37A  D697N  G757R  G178E  S359F  E778K  K768E  E590K  Y834N  D242N  D576N  R116W  D584N  W771  L152F  R735C  R735C  G339R  G356E  E810K  P577S  H370N  E819K  H423N  E762K  P509H  D167N  R793  E318K  R542Q  R153L  R153L  Q255  P417L  P401S  W170  E211  L157F  H658Q  G633V  L299I  V217F  X629_splice  G727E  X402_splice  E86  W243L  D836H  T831I | Missense_Mutation  Nonsense_Mutation  Missense_Mutation  Missense_Mutation  Missense_Mutation  Missense_Mutation  Missense_Mutation  Missense_Mutation  Missense_Mutation  Missense_Mutation  Missense_Mutation  Missense_Mutation  Missense_Mutation  Missense_Mutation  Missense_Mutation  Missense_Mutation  Missense_Mutation  Missense_Mutation  Missense_Mutation  Nonsense_Mutation  Missense_Mutation  Missense_Mutation  Missense_Mutation  Missense_Mutation  Missense_Mutation  Missense_Mutation  Missense_Mutation  Missense_Mutation  Missense_Mutation  Missense_Mutation  Missense_Mutation  Missense_Mutation  Missense_Mutation  Nonsense_Mutation  Missense_Mutation  Missense_Mutation  Missense_Mutation  Missense_Mutation  Nonsense_Mutation  Missense_Mutation  Missense_Mutation  Nonsense_Mutation  Nonsense_Mutation  Missense_Mutation  Missense_Mutation  Missense_Mutation  Missense_Mutation  Missense_Mutation  Splice_Region  Missense_Mutation  Splice_Region  Nonsense_Mutation  Missense_Mutation  Missense_Mutation  Missense_Mutation | SNP  SNP  SNP  SNP  SNP  SNP  SNP  SNP  SNP  SNP  SNP  SNP  SNP  SNP  SNP  SNP  SNP  SNP  SNP  SNP  SNP  SNP  SNP  SNP  SNP  SNP  SNP  SNP  SNP  SNP  SNP  SNP  SNP  SNP  SNP  SNP  SNP  SNP  SNP  SNP  SNP  SNP  SNP  SNP  SNP  SNP  SNP  SNP  SNP  SNP  SNP  SNP  SNP  SNP  SNP | unknown significance  unknown significance  unknown significance  unknown significance  unknown significance  unknown significance  unknown significance  unknown significance  unknown significance  unknown significance  unknown significance  unknown significance  unknown significance  unknown significance  unknown significance  unknown significance  unknown significance  unknown significance  unknown significance  unknown significance  unknown significance  unknown significance  unknown significance  unknown significance  unknown significance  unknown significance  unknown significance  unknown significance  unknown significance  unknown significance  unknown significance  unknown significance  unknown significance  unknown significance  unknown significance  unknown significance  unknown significance  unknown significance  unknown significance  unknown significance  unknown significance  unknown significance  unknown significance  unknown significance  unknown significance  unknown significance  unknown significance  unknown significance  unknown significance  unknown significance  unknown significance  unknown significance  unknown significance  unknown significance  unknown significance |

TCGA, The Cancer Genome Altlas; SKCM, skin cutaneous melanoma; SNP, single nucleotide polymorphism.

**Supplementary Table S2.** Data sources.

| **Cohort** | **Tumor** | **Treatment** | **N** | **Sequencing** | **Clinical outcome** | **Reference** |
| --- | --- | --- | --- | --- | --- | --- |
| VanAllen15  Riaz17  Miao18  Liu19  MSKCC  TCGA-SKCM | Melanoma  Melanoma  Melanoma  Melanoma  Pan-cancer  Melanoma | Anti-CTLA-4  Anti-PD-(L)1  Anti-CTLA-4/Anti-PD-(L)1/Combined  Anti-PD-(L)1  Anti-CTLA-4/Anti-PD-(L)1/Combined  - | 110  68  132  125  1661  440 | WES  WES  WES  WES  NGS panel  WES | ORR, DCR, OS  ORR, DCR, OS  ORR, DCR, OS  ORR, DCR, OS  OS  OS | Science. 2015;350(6257):207-211.  Cell. 2017;171(4):934-949.e16.  Nat Genet. 2018;50(9):1271-1281.  Nat Med. 2019;25(12):1916-1927.  Nat Genet. 2019;51(2):202-206.  Cell. 2018;173(2):291-304.e6. |

TCGA, The Cancer Genome Altlas; SKCM, skin cutaneous melanoma; WES, whole-exome sequencing; NGS, next generation sequencing; ORR, objective response rate; DCR, disease control rate; OS, overall survival.
